# Development and randomized controlled trial evaluation of E-learning trainings for professionals

**DOI:** 10.1186/s13690-020-00465-4

**Published:** 2020-11-23

**Authors:** Elisa König, Anna Maier, Jörg Michael Fegert, Ulrike Hoffmann

**Affiliations:** Department of Child and Adolescent Psychiatry/Psychotherapy, University Hospital of Ulm, Steinhoevelstr. 5, 89075 Ulm, Germany

**Keywords:** Child protection, Child maltreatment, Continuing medical education, E-learning, Health professionals, Safeguarding standards

## Abstract

**Background:**

Child maltreatment and consequently child protection are highly relevant and current issues in our society. Medical institutions are widely regarded as places of healing, care and support. But they also hold risk factors to promote child maltreatment. Efforts have to be taken in order to offer help to victims by medical institutions and to reduce risk factors for child maltreatment. Therefore, health professionals in the field of child protection must be trained and sensitized for these two purposes.

The Department of Child and Adolescent Psychiatry / Psychotherapy at the University Hospital of Ulm in Germany is developing E-Learning courses directed to health professionals in order to create flexible advanced training courses for dealing with child abuse, and to increase competences in child protection. Due to their specific role in (institutional) child protection, three courses and their evaluation will be presented in this article. The aim of the studies is to examine if those online-courses are increasing knowledge and skills in child protection and how satisfied participants are with course quality.

**Methods:**

Randomised Controlled Trials (RCT) were conducted with one wait-list control group and one group participating in the course (= intervention group). The RCTs took place from October 2016 to March 2017 for two courses, and from May 2017 to September 2017 for the other course. Data were analysed with mixed design ANOVA. For evaluation of user satisfaction, descriptive statistics are reported.

**Results:**

For all three courses, knowledge and practical capacities on the topic of the intervention group raised significantly in comparison to the values of the control group. Furthermore, participants of the course for managers felt better prepared to meet their responsibilities in regard to institutional child protection and came up with ideas on how to implement safeguarding standards in their institution. Overall, participants were very satisfied with the structure and the content of the courses.

**Conclusions:**

The article shows that the online-courses are an effective and well-accepted approach to train professionals in topics regarding (institutional) child protection by contributing to the participants´ abilities to create medical facilities into a place of competence and protection.

## Background

In their sustainability goals, the United Nations demands that maltreatment, exploitation, trafficking and all forms of violence and torture against children must end by the year 2030 (goal 16.2) [1]. In Europe, tens of millions of children and adolescents are affected by various forms of child maltreatment. The World Health Organization (WHO) estimates that 90% of these cases are overlooked by professionals in institutions [2].

In Germany, there is a constant high prevalence of child maltreatment (neglect, physical and emotional maltreatment and child sexual abuse) [3]. Nevertheless, the German society and politics aren’t sufficiently aware of the importance of child protection yet [2, 3]. In addition, experiences of maltreatment in childhood and adolescence are a risk factor for psychological and somatic secondary diseases and social consequences [4]. Child maltreatment is thus one of the main causes of health inequality and social injustice [2]. This results in high social costs, including high burdens on the health care system [5]. Therefore, child maltreatment is not only an enormous challenge for those affected and their relatives, but also a burden for public health and the health system.

Consequently, it is crucial to find ways to enable children to grow up without violence or to recognize maltreatment at an early stage and to provide adequate support. Only in this way can serious long-term consequences be prevented and the health of children and adolescents be promoted. An important component in recognizing and preventing child maltreatment is education and raising awareness of the issue by building up knowledge and capacities among health professionals, as they are central actors in child protection through their regular contact with children and adolescents.

Medical institutions are widely regarded as places of healing, care and support. With regard to child maltreatment, they must be a place of both competence and protection.

A medical institution that is seen as a place of competence offers competent help and support to children and adolescents affected by child maltreatment (often occurring in family-context). As a place of protection, the institution implements structures, measures and procedures enabling the recognition, identification and prevention of assaults against children. Health professionals in the field of child protection must therefore be trained and sensitized for these two purposes.

So far in Germany, the topic of (institutional) child protection has not been included in the education, training and further education of health care professionals at all or only to a limited extent [6]. Additionally, the WHO demands that the “prevention of child maltreatment needs to be mainstreamed into the curricula of health and other professionals” [2]. E-Learning is one approach in offering training to professionals. E-Learning holds several advantages: the facilitation of combining career and family life, flexibility, possibility to offer training nation-wide and to keep content up-dated.

In the Department of Child and Adolescent Psychiatry / Psychotherapy at the University Hospital of Ulm in Germany, child protection has been a major focus since the clinic was founded almost 20 years ago. In the last 10 years, one research section specialized in developing and evaluating E-Learning courses in numerous projects. Some of these courses are directed to health professionals and focus on topics regarding (institutional) child protection. Due to their specific role in (institutional) child protection, the following three projects and their evaluation will be briefly presented here:

- Child Protection in Medicine - a basic course for all health professionals [7]
- Safeguarding standards in institutions [8]
- Basic knowledge of child protection in institutions – an online course for managers [9]

The first online-course can be seen as training that enhances the competences of professionals working in institutions that support affected children by qualifying their employees in child protection topics and raising awareness among them. The two latter courses aim to strengthen institutions by implementing safeguarding standards and creating a safe and protected atmosphere for children. This can be achieved by qualifying employees accordingly.

All three online-courses were evaluated to prove if course participation increases participants´ knowledge and skills in child protection using an RCT design. We hypothesized that knowledge and skills in child protection would rise significantly more in the intervention group compared to the wait-list control group. Another scope of course evaluation was to explore user satisfaction with the online-courses.

## Methods

### Online-courses

Learning objectives of all courses comprise transfer of knowledge and action competence along with emotional learning. As previous research and reports showed, the private or institutional environment of the victims often ignored hints and statements of abused children. Therefore, a development of training programs on child protection does not only aim at transferring knowledge and action competence, but also at sensitizing to the needs of victims in the sense of establishing a “culture of attentiveness” in regard to the needs of children and adolescents.

**Fig. 1:**
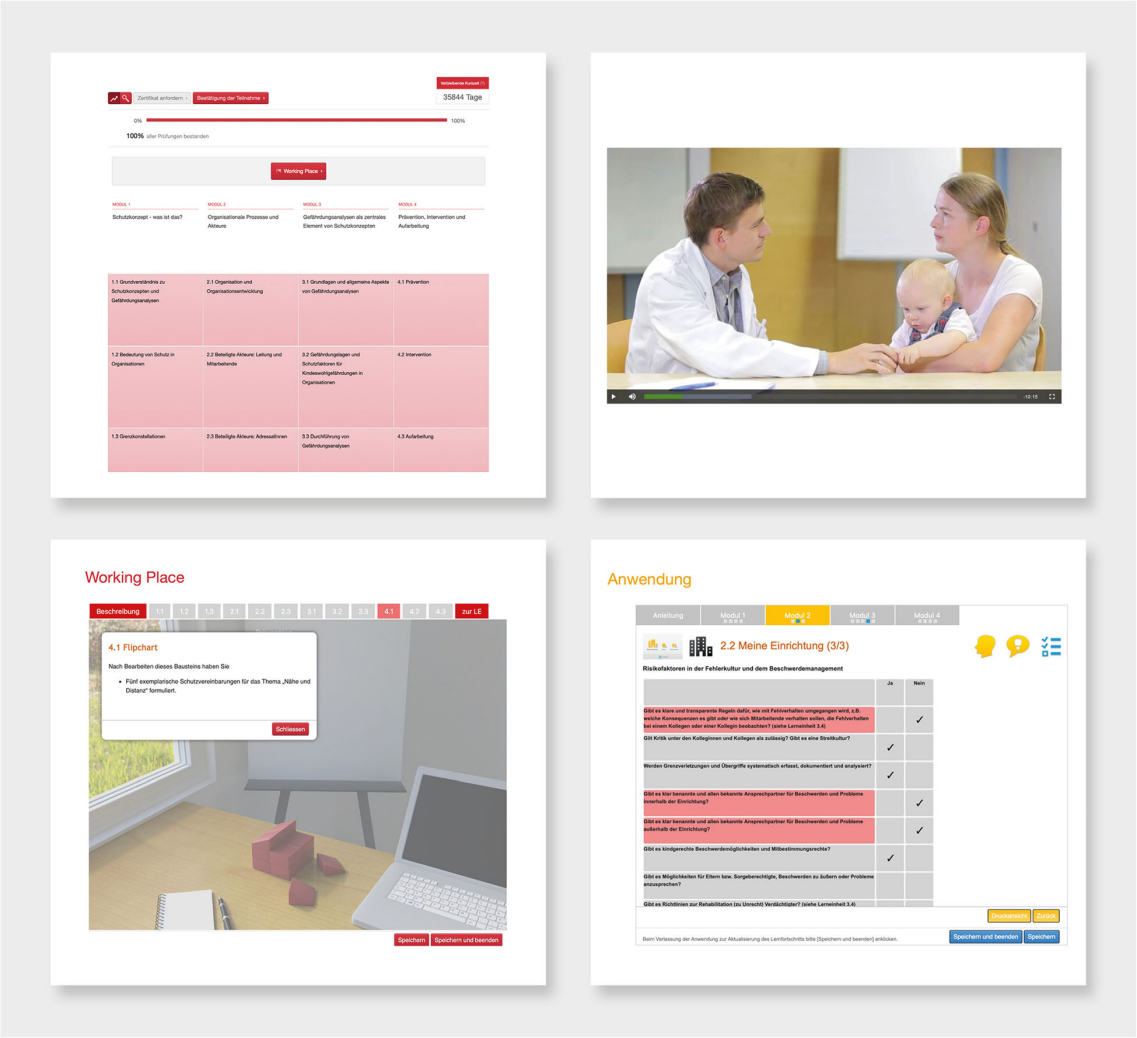
Screenshots of interfaces of online-courses. Top left: example of diagram of the modules, top right: example of a good-practice video-based role-play, bottom left: example of working place to transfer knowledge to the own work-setting, bottom right: example of check-list of analyzing institutional structures

To reach the learning objectives, the online-courses comprise a broad range of didactical methods like text-based materials, case-studies, video-clips and download materials like check-lists and templates (see Fig. 1).

All online courses have a modular structure, i.e. there are several learning units, which are categorized to comprehensive modules. In the following, the three online-courses will be described in detail.

#### Child protection in medicine - a basic course for all health professions

The online course has been developed and evaluated since June 2015. The aim of the online course is to provide health professionals an overview of epidemiology and diagnostics of the forms of child maltreatment, the current legal situation and the legal regulations. Target group of the course are all health professionals. The course contains five modules in the theoretical section with a total of 18 learning units.

In addition to imparting knowledge through basic principles and legal texts, the course places particular importance on case-based learning. In the practical section, ten case studies from different medical fields are offered for processing. The cases reflect different forms of child maltreatment and vary in factors such as the age of the child and family constellation, so that a wide range of cases that actually occur in practice is covered. In addition, film clips on how to conduct a conversation in a child protection case and exercises on the topic are available.

The course has been developed and revised since 2015. It takes about 30 h to complete the course. In sum, 1960 individuals accomplished the course successfully until now. The online course cooperates closely with the German Child Protection Hotline team. The German Child protection Hotline is a free 24-h advisory service for health professionals, and it continually expands the content of the course to include relevant topics that arise at the Child Protection Hotline. This cooperation was highlighted in the latest report on preventing child maltreatment of the WHO as a lighthouse project [5].

#### Safeguarding standards in institutions

In 2010, sexual abuse in German institutions of the Catholic Church and reform education became public. As a consequence, in the public and scientific community the question as to how institutional structures, procedures and standards should be conceptualized to prevent assaults against children and how to improve support for affected children, gained importance. Committees were politically installed to explicitly discuss the question, like the round table on sexual abuse, in the context of dependency and power in private and public institution and in families. It was no longer possible to declare those affected institutions as single cases, but to understand, that every institution holds potential risk factors for child maltreatment. Therefore, all institutions directly engaging with or providing services to children need to ensure that they constitute a child safe environment. Safeguarding standards aim at reducing such risk factors, preventing child maltreatment and fostering a child safe environment in institutions. Examples for such standards a mission statement, a code of conduct or a comprehensive complaint system. Standards should be concluded for each institution specifically from an institutions risk analysis, identifying situations and procedures at high risk for child maltreatment. The goal of the online course “safeguarding standards in institutions” is to support organizations in this process of identifying and implementing necessary safeguarding standards. The course was developed and revised in 2014 and comprises four learning modules with a total of 12 learning units. It takes about 35 h to complete the course. The course is certified with 40 credit-points by the Medical Association. Topics of the course include the structure and the development of safeguarding standards, with a special consideration towards particular characteristics of the institution and the enhancement of awareness for the topic. Different methods of conducting a risk analysis and standards of prevention, intervention and inquiry are presented. In sum, 346 individuals accomplished the course successfully until now.

#### Basic knowledge of child protection in institutions – an online course for managers

As those holding institutional leadership have a crucial position and are particularly responsible for implementing safeguarding standards, a specific training for managing positions has been developed since 2014 including a broader context regarding the implementation of safeguarding standards such as basic information about child maltreatment, organizational structures and culture, staff management/ human resource, change management and relevant legal aspects, e.g. labour law. The 34-h-curriculum has 16 learning units categorized into 4 learning modules. It is certified with 60 credit points. The objective is to enable and support participants in creating an institutional context which minimizes the opportunity for maltreatment to occur and embeds child safety in the institutional culture. Up to date, a total of 380 individuals completed the course with a certificate.

### Participants

Target group of the online-course and the RCT-study “Child protection in medicine” was medical staff (physicians, (psycho) therapists, nurses). The online-course and conjoined study “Safeguarding standards in institutions” aimed at employees of institutions holding responsibility for children and adolescents (like schools, institution of youth welfare etc.). The online-course for managers addressed people in managing positions of such institutions.

Participants were recruited in Germany by utilizing mailing lists of associations and societies in the field, presentations at congresses and publications in journals.

After eligible participants were enrolled in the study, they were randomly assigned to an intervention group and a wait-list control group using computer generated random sequences.

The RCT study of the online-course “Child protection in medicine” comprised *N* = 262 participants in the intervention group and *N* = 228 participants in the wait-list control group. The total number of participants of the online-course “Safeguarding standards in institutions” was 190 (80 in the intervention group and 110 in the wait-list control group). Two hundred forty-three persons participated in the RCT study of the online-course for managers with *N* = 136 in the intervention group and *N* = 107 in the wait-list control group.

Table 1 summarizes demographics separated by online-courses and groups. The intervention group and the wait-list control group did not differ significantly in any of the three online-courses regarding age, gender, profession and work experience.

**Table 1:** Demographic data of the samples, separated by online-courses and groups

	Child protection in medicine		Safeguarding standards in institutions		Course for managers	
	IG (*N* = 262)	CG (*N* = 228)	IG (*N* = 80)	CG (*N* = 110)	IG (*N* = 107)	CG (*N* = 136)
Age, M (SD)	42.3 (9.9)	42.4 (10.0)	40.6 (10.1)	39.5 (11.3)	45.4 (8.6)	47.1 (9.0)
Gender, % female	85.9	84.2	86.3	83.6	76.6	69.9
Work experience, years M (SD)	13.7 (10.2)	12.7 (10.2)	10.6 (9.2)	10.7 (9.6)	15.7 (8.6)	17.6 (9.8)

*IG* Intervention group, *CG* Wait-list control group, *M* Mean, *SD* Standard Deviation, *N* Sample

The intervention group and the wait-list control group did not differ significantly in any of the presented demographic data.

### Procedure

The study was conducted according to a wait-list control group design with an intervention group, and a wait-list control group which received the same online-course as the intervention group about 5 months after the intervention group started. After randomization, participants of the intervention group completed pre-assessment before course-start (T1), which was repeated after online-course (T2). At matched time-points the wait-list control participants completed the baseline-assessment (T1) and the pre-assessment before course-start (T2). All assessments were conducted using online questionnaires. The RCT of the online-course “child protection in medicine” took place from May 2017 until September 2017, the RCTS of the other two courses about safeguarding standards and child protection in institutions were conducted from October 2016 until March 2017.

Participation in the online-courses was free of charge. Participants were free to complete the online course at their own pace just so long as it was completed within 6 months. All courses got certified by the Medical Association of the federal state Baden Württemberg within the Continuing Medical Education (CME) system. Table 2 offers an overview of the key characteristic of the three online-courses.

**Table 2:** Key points of the three online-courses described

Online-Course	Modules (Number of learning units)	Target of the course	Processing time in hours (CME-Points)	Sponsor
Child protection in medicine - a basic course for all health professions	Forms of Maltreatment, Theoretical Basics, Practical Basics (18)	Provide health professionals an overview of the epidemiology and diagnostics of the forms of maltreatment and legal regulations.	30 (36)	German Federal Ministry of Health
Safeguarding standards in institutions	Definition of institutional safeguarding standards, Organizational procedures and agents, Risk analyses, Prevention, Intervention and Inquiry (12)	Support participants in analyzing potential risks for assaults against children and developing safeguarding standards in their institution.	35 (40)	Federal Ministry of Education and Research
Basic knowledge of child protection in institutions – an online course for managers	Introduction/Basics, Institutional risk and protective factors; Staff management/HR; Implementation of safeguarding standards (16)	Managers in leading positions are to be enabled to actively shape the development of safeguarding standards, to create a trauma-sensitive environment in the institutions and to be aware of relevant legal aspects in this context.	34 (60)	Federal Ministry of Education and Research

*CME* Continuing Medical Education

### Measures

Data collection at T1 and T2 included the assessment of perceived knowledge on the topic (item 1) and perceived practical capacities regarding the topic (item 2) on 6-point-likert scale (1 = very little to 6 = very extensive). To add an objective evaluation of gained competences, in each course a self-administered multiple-choice-test comprising 20 to 30 items with 5 options each was conducted.

Regarding the courses for managers, we were interested whether course participation changed the perspective on one’s own role in the context of institutional child protection. Therefore, eleven items were assessed at T2 assessment on a 6 point-likert scale measuring affirmation with the given statements (1 = don’t agree at all to 6 = agree completely).

Another goal was to assess participants´ satisfaction with the course, such as users´ satisfaction with learning materials or structure of the course to evaluate acceptance and get hints for further revision. Therefore, participants who successfully completed the course assessed the quality of the course at the end (T2). The evaluation of user satisfaction with the online-courses included items assessing the content, structure, and materials.

If not indicated differently, the reported items have a 6-point-likert-scale (1 = I don’t agree at all to 6 = I agree completely).

In the following, results for the sample of the RCT will be reported. Further details on the evaluation design of the online-course can be found in other publications [10, 11].

### Statistical analysis

Statistical analyses were performed using the Statistical Package for the Social Sciences SPSS 23.0. In order to examine the effect of the online-courses on knowledge and practical capacities, mixed-design ANOVAs were applied using the groups (intervention group and control group) as a fixed factor and time point as a within-participants repeated factor.

The study was approved by the institutional review board of the University of Ulm.

## Results

### Effectiveness of online courses

For all three courses, the subjective estimation of participants of the intervention group regarding their knowledge and their practical capacities on the topic raised significantly in comparison to the values of the control group. This is reflected in the results of the multiple-choice-test: knowledge of the intervention group improved significantly compared to the control group. Specific numbers are presented in Table 3.

**Table 3:** Pre-post group analysis for evaluation of efficacy for all three online-courses

	Control group		Intervention group		F-Value of time x group
	T1 M (SD)^a^	T2 M (SD)^b^	T1 M (SD)^c^	T2 M (SD)^d^	
	***Child protection in medicine***				
	*N* = 228		*N* = 262		
Knowledge [Score]^e^ (8 Items)	24.6 (7.2)	36.3 (4.9)	26.8 (6.8)	35.9 (5.5)	F (1) = 17.1***
Practical Capacities^f^	3.2 (1.0)	4.5 (0.7)	3.4 (0.9)	4.5 (0.7)	F (1) = 8.3**
	*N* = 316		*N* = 262		
Score in MC-Test in % (123 Items)	73.9 (6.0)	74.8 (8.6)	74.4 (6.7)	86.8 (7.0)	F (1) = 28.6***
	***Safeguarding standards in institutions***				
	*N* = 110		*N* = 80		
Knowledge^f^	3.2 (0.9)	3.0 (1.0)	3.0 (1.1)	4.3 (0.7)	F (1) = 127.8***
Practical Capacities^f^	3.3 (1.0)	3.1 (1.1)	3.2 (1.0)	4.1 (0.9)	F (1) = 61.6***
Score in MC-Test in % (21 items)	68.6 (6.8)	68.5 (7.3)	68.6 (7.0)	76.2 (8.5)	F (1) = 47.0***
	***Course for managers***				
	*N* = 136		*N* = 107		
Knowledge^f^	4.0 (0.8)	4.0 (0.8)	4.0 (0.8)	4.5 (0.7)	F (1) = 23.7***
Practical Capacities^f^	4.0 (0.8)	4.0 (0.7)	3.9 (0.9)	4.5 (0.8)	F (1) = 19.0***
Score in MC-Test in % (30 items)	73.6 (6.8)	74.6 (6.6)	72.2 (6.9)	80.1 (8.0)	F (1) = 100.8***

M *Mean*, *SD* Standard Deviation, *N* Sample, *MC* Multiple Choice, ^a^Baseline-assessment, ^b^Assessment 6 months after baseline-assessment, ^c^Pre-assessment before course-start, ^d^Post-assessment after course has been finished, ^e^Min: 8, Max: 48, ^f^Min: 1, Max:6.

* indicates significane level of statistcial analyses with * *p* < .05; ** *p* < .01; *** *p* < .001

Participants of the course for managers were asked if they gained a better understanding of their role in the context of institutional child protection by course participation. The majority of the 199 managers who have finished the course within the RCT study affirmed, that by course participation it is clearer to them which safeguarding standards still have to be improved (88%) and what their tasks and duties are in context of institutional child protection (84%), they feel better prepared to meet their responsibilities as managers in regard to institutional child protection (86%) and they came up with ideas/suggestions on how to implement safeguarding standards in their institution (85%).

### Evaluation of user satisfaction with the online-courses

The mean scores of different aspects concerning users´ satisfaction with structure and content of the courses are high (see Table 4): Participants were satisfied with navigation, structure of content and the learning materials. They stated that the learning material is relevant to their professional work and that E-learning is a suitable method for conveying information on the topic. The majority of participants (64–87%) stated that the standard of content and the depth of information was appropriate.

**Table 4:** Mean and standard deviation of items assessing user satisfaction for all three courses

Item	Child protection in medicine M (SD); *N* = 490	Safeguarding standards in institutions M (SD); *N* = 148	Course for managing positions M (SD); *N* = 199
I was well orientated on the website.	5.5 (0.8)	5.0 (1.0)	5.3 (0.9)
The structure of the training was coherent.	5.4 (0.8)	5.3 (0.9)	5.2 (0.9)
I was pleased with the learning materials.	5.1 (0.8)	5.2 (0.9)	5.2 (0.8)
The learning content is relevant for my professional activities.	5.1 (1.1)	5.0 (1.1)	5.3 (0.9)
E-learning is an appropriate form of advanced training regarding the issue.	5.2 (0.9)	5.1 (1.0)	5.2 (0.9)
The expenditure of time I spent for the course was profitable.	5.5 (0.9)	5.3 (1.0)	5.3 (0.9)

*M* Mean, *SD* Standard deviation, *N* Sample

## Discussion

While most cases of child maltreatment take place in an intra-familial environment, institutions working with children have an important part in protecting children in two ways: firstly, by providing maltreated children and adolescents a place of competence offering help, support and treatment, if necessary. Those affected often seek help in an institutional medical context, this means that cases of child maltreatment become visible for the first time in medical institutions. Although there is sometimes little time to pursue suspicions in these cases, it is very important that health professionals react adequately to suspicions of child maltreatment. Secondly, institutions play an important role in protecting children, because the nature of medical institutions implies numerous risk factors which promote child maltreatment, by implementing safeguarding standards, thus cases of child maltreatment can be recognized, identified and ideally prevented earlier. Such risk factors comprise intimate nursing situations, personal one-to-one settings like in psychotherapy, or inhibited conscious states due to medication. In addition, particularly in medical institutions, there are often steep hierarchies which make it more difficult to deal with cases of child maltreatment adequately. Several studies showed that medical institutions can act as crime scenes in which professionals misuse structures and treatment situations to abuse their patients [12]. This perspective is often neglected in the self-image of medical institutions as it contradicts the self-perception of medical institutions as places where people get help and treatment.

For both cases, i.e. dealing with intra-familial cases that became apparent in the medical institution as well as intra-institutional cases, there are many uncertainties among professionals and medical institutions on how to proceed and how to take preventive measures [2]. In order to counter this challenge, it is crucial to offer continuing medical education for health professionals on (institutional) child maltreatment, because child maltreatment is not only a concern for the individuals, but an issue that affects society as a whole, and in particular the public health sector. Medical facilities must be both places of competence and protection for children and adolescents affected by child maltreatment.

Our research shows that the online-courses are an effective and well-accepted approach in order to train professionals in topics of (institutional) child protection, by contributing to the participants´ abilities to create medical facilities as places of competence and protection. E-Learning offers the possibility of a flexible qualification with respect to time and location making it a good learning method to combine career and family life. It enables individualized learning and it can easily be provided nation-wide. That becomes evident in the current situation in context of the COVID-19 pandemic, as face-to-face training had to be cancelled or individuals had to be in quarantine. At the same time, the topics of child protection and domestic violence came into focus in the public and professional discussion [13]. Subsequently, a high demand for evaluated and quality E-Learning trainings arose. It is shown in the number of registrations for the online-courses offered by Ulm University (see https://elearning-kinderschutz.de/ to find information about present online-courses offered by Ulm University and possibilities of participation). For example, between the 11th of March 2020 and 12th of May 2020 (63 days), 1447 individuals registered for the online-course “child protection in medicine”, compared to 600 individuals during the same time span (08th January 2020 until 10th March 2020) before actions and decision were taken in Germany on a political level to curtail COVID-19.

A limitation to this work is that the data are not based on a representative data set, because the participants of the online courses could not be recruited systematically and thus there is probably an overrepresentation of professionals who are particularly interested or committed to the topic of the online course in the sample. Furthermore, no statements can be made about the long-term effects of the measures, since the surveys are only momentary snapshots.

Despite the limitations mentioned above, the results provide important insights into the need and opportunities for further training in the area of child protection among health professionals.

## Conclusions

Summing up, E-Learning seems to hold the potential to act as an important component for improved child protection and to close existing and newly risen gaps in further training about child protection for professionals working with children. Corresponding E-Learning calls for or seeks for other European countries to be considered in order to address the comprehensive problem of child maltreatment across the whole of Europe.

## Data Availability

The datasets generated and/or analyzed during the current studies are not publicly available due to reasons of data security, but are available from the corresponding authors on reasonable request.

## Abbreviations

CME: Continuing Medical Education
M: Mean
MC: Multiple Choice
N: Sample
RCT: Randomised Controlled Trial
SD: Standard Deviation
WHO: World Health Organization
